# Antipsychotic medication non-adherence and factors associated among patients with schizophrenia in eastern Ethiopia

**DOI:** 10.1186/s12888-024-05554-0

**Published:** 2024-02-07

**Authors:** Fethia Mohammed, Biftu Geda, Tesfaye Assebe Yadeta, Yadeta Dessie

**Affiliations:** 1 Department of Psychiatry, School of Nursing and Midwifery, College of Health and Medical Sciences, Haramaya University, Harar, Ethiopia; 2 Department of Nursing, School of Health Sciences, Madda Walabu University, Shashamane Campus, Shashamane, Ethiopia; 3 School of Nursing and Midwifery, College of Health and Medical Sciences, Haramaya University, Harar, Ethiopia; 4 School of Public Health, College of Health and Medical Sciences, Haramaya University, Harar, Ethiopia

**Keywords:** Antipsychotic, Schizophrenia, Substance use, Non-adherence, Factor, Ethiopia

## Abstract

**Background:**

Given that antipsychotic medication is a cornerstone for treating and preventing relapse in people with schizophrenia, non-adherence has been indicated as a big challenge. This study aimed to assess antipsychotic medication non-adherence and factors associated among patients with schizophrenia in eastern Ethiopia.

**Methods:**

We conducted an institution-based cross-sectional study in two public hospitals in Eastern Ethiopia from December 1, 2022, to January 31, 2023. Antipsychotic medication adherence was assessed using MOrisky medication adherence rating scale questionnaire, and insight was measured using the self-report insight scale for Psychosis (ISP). Multiple stepwise logistic regression models with Adjusted Odds Ratio (AOR) and 95% confidence interval (CI) were applied to identify the factors. Statistical significance was considered at p-value ≤ 0.05.

**Results:**

We found that 44.57% of patients with schizophrenia experienced non-adherence to their antipsychotic medication. Being single (AOR = 2.48, 95% confidence interval [CI]:1.71, 3.58), alcohol users (AOR = 2.00, 95% confidence interval [CI]:1.46, 2.72), Khat chewers (AOR = 2.84, 95% confidence interval [CI]; 2.06, 3.90) and having no insight to their illness (AOR = 2.1, 95% confidence interval [CI]:1.52, 2.90) were more likely to be non-adherent to their antipsychotic medications.

**Conclusions:**

Our study revealed that antipsychotic medication non-adherence was high among individuals suffering from schizophrenia and that it was influenced by various factors such as single marital status, alcohol usage, Khat chewing, and having no understanding of their condition. As a result, comprehensive intervention methods should be developed to address the factors associated with psychotropic medication non-adherence among patients. Healthcare professionals should pay attention to these aspects and consider developing specific strategies to promote adherence to medications while treating individuals with schizophrenia.

## Introduction

Schizophrenia is among the ten top diseases that contribute to global disease burden requiring a long-term antipsychotic medication treatment [1]. For people experiencing the problem, medication is a cornerstone [2] and as it helps to reduce psychotic symptoms, and improving psychosocial functioning when there is a proper adherence [3]. In this sense, antipsychotic medication adherence refers to patients’ ability to take their prescribed medications as recommended by their physicians [4] and is essential to improve symptoms and lower relapse rates(5). Whereas, non-adherence to medication, on the other hand, is among the most serious problem, component of the treatment [6]. Many patients with schizophrenia experience medication non-adherence at some point during their treatment, putting them at a significantly higher risk of illness exacerbation, relapse, re-hospitalization, or increased use of emergency psychiatric services, functional decline, and an increased risk of death, as well as increasing the care burden on their families [3, 7].Studies have indicated that medication non-adherence among schizophrenia patients ranges between 56% and 60% [8, 9], with relapse rate from 75 to 90% [10]. Studies have shown that patients with schizophrenia who receive treatment at specific mental health facilities have a higher risk of relapse due to non-adherence to antipsychotic medication [2, 11], resulting in higher rates of hospitalization [12].

Antipsychotic medication non-adherence among patients with schizophrenia has been associated with several factors such as unemployment, educational status [13], age, gender, living in rural areas, having a short duration of the disease [13–17], misunderstanding the diagnosis, substance abuse, self-stigma, a lack of social support, and a family history of mental illness [5, 8, 18]. Other factors include side effects from antipsychotic medications, an inadequate supply of antipsychotic medications [19], a preference for alternative medicine [20], the distance between the treatment center and the patient’s home, treatment dissatisfaction, the patient’s level of insight into their illness, or their attitude toward antipsychotic medication.

a [21].

Additionally, a qualitative study conducted in rural Ethiopia found that a number of factors influence medication adherence, such as substance abuse, food insecurity, stigma, a lack of understanding, a lack of family or social support, an inability to recover with antipsychotic treatment, side effects from medication, and dissatisfaction with health workers’ approaches [22].

. A systematic review and meta-analysis study showed that chewing “Khat” was associated with non-adherence to antipsychotic medication among patients with schizophrenia [8]. Data regarding antipsychotic medication non-adherence and factors associated with patients with schizophrenia in eastern Ethiopia, particularly from the current study area, is scarce. As a result, identifying antipsychotic medication non-adherence and factors associated among patients with schizophrenia in eastern Ethiopia will aid in the development of intervention strategies and programs to address this issue in Ethiopia. Therefore, the purpose of this study was to assess antipsychotic medication non-adherence and factors associated among patients with schizophrenia in eastern Ethiopia.

## Methods and materials

### Study setting

This study was conducted at Hiwot Fana Comprehensive Specialized University Hospital, located in Harar town, and Dil-Chora Referral, located in Dire Dawa City administration, both in eastern Ethiopia. Hiwot Fana Comprehensive Specialized University Hospital is located in Harar town. Harar is the capital city of Harari Regional State, located in eastern Ethiopia at a distance of 526 km from Addis Ababa. There are two public hospitals in the Harari Region (Hiwot Fana Specialized University Hospital and Jogul Regional Referral Hospital). Hiwot Fana Specialized University Hospital was established in 1941 and became a university-specialized hospital in 2010. It provides service to more than 5.8 million people in its catchment area of eastern Ethiopia, of whom more than 10,000 are psychiatric patients. Hiwot Fana Comprehensive Specialized University Hospital has three outpatient departments (OPD), one psychiatry emergency, 12 inpatient beds (7 beds were allocated to male patients, 4 beds for females, and one bed was assigned to a neuropsychiatric patient), and three outpatients with one psychiatrist. There are two psychiatrists, three master’s degree holders in clinical and community mental health professionals, and five first-degree psychiatry nurses.

DilChora Referral Hospital is located in Dire Dawa city administration, which is 515 km from Addis Ababa. It is the only referral hospital in the city and was established in 1960. DilChora Referral Hospital, located in the northeast of the city, provides service for more than 45,000 people in the catchment area of eastern Ethiopia, of whom more than 6,000 are psychiatry patients. DilChora Referral Hospital has two outpatient departments (OPD), no psychiatry emergency room, and six patient beds, of which three are allocated for male patients and three are for female patients. There are two master degree holders in clinical and community mental health professionals, three first-degree psychiatry nurses, and one psychiatrist. The study was conducted from December 1, 2022, to January 31, 2023.

### Population

Study population was patient who diagnosed with schizophrenia on follow-up as outpatients at the psychiatry department of the hospitals for the last 6 months at both hospitals.

### Inclusion and exclusion criteria

The inclusion criteria were patients whose age was 18 years and above who were diagnosed with schizophrenia and had been on follow-up as outpatients at the psychiatry department of the hospitals and on antipsychotic medication for at least six months prior to the data collection period and volunteered to give consent were included.

Exclusion Criteria Patients with schizophrenia who had received outpatient care at the hospital psychiatry department and had been followed up as outpatients for less than six months prior to the data collection period, as well as those who had acute symptoms at the time of data collection, were excluded.

### Study design

Institution based cross-sectional study was conducted in public hospital of Eastern Ethiopia from December 1, 2022, to January 31, 2023.

#### Sample size determination

The sample size was calculated using open epi online software considering the following assumptions OR = 1.5 [23], 80% power of the study, 95% confidence interval, and 10% non-response rate, the estimated final sample size were 911.

### Variables of study and measurements

#### Dependent variable

Antipsychotic medication non adherence.

### Data collection

#### Independent variables

Socio-demographic variables (sex, age in years, marital status, occupational status, educational status, residence); clinical factors (family history of mental illness, insight to illness and medication related side effect), substance-related factors (current and lifetime substance use of alcohol, tobacco, Khat, and cannabis/ hashish).

#### Data collection instruments

A structured questionnaires was used to obtain information about Socio-demographic characteristics and clinical factors. Substance-related factors were assessed by the Alcohol, Smoking, and Substance Involvement Screening Test(ASSIST), which is a brief screening questionnaire developed and validated by the world health organization (WHO) to find out about people’s use of psychoactive substances use to assess current and ever substance use history of the subject [24]. Antipsychotic medication adherence, was measured using a modified version of, Medication Adherence Rating Scale (MARS), which is a 10-item self-report scale, was used. The item answered with a yes/no response as well as a 0 or 1 value. A MARS score of 3 or higher indicates adherence, while a MARS score of less than or equal to 2 indicates non-adherence to the antipsychotic treatment [25]. Insight was measured using the self-report insight scale for Psychosis( ISP) is an 8-item self-report scale that is simple to answer and represent three subscales: awareness of illness (2 items), symptom relabeling (2 items), and treatment need (4 items). Has been shown to have good reliability and validity in people who experience psychotic symptoms each subscale has a total score ranging from 0 to 4. With a score of 9 or more indicating good insight [26].

Antipsychotic side effects was assessed using Glasgow Antipsychotic Side effect Scale (GASS) which contains the 22-item. The extent of side effects was rated from none (0) to everyday (3 points) for the first 20 questions and no (0) and yes (3 points) for the last two questions. Patients with a total score of 0–21 indicated absent/mild side effects, those scored 22–42 indicate moderate side effects, and those recorded 43–63 showed to be severe side effects [27].

### Data quality control

Data collectors and supervisors were trained for three days on the data collection approach of the study. The questionnaire was translated into local language, Afan Oromo and Amharic, by language experts and back-translated into English by another person to check for consistency. A pretest was conducted on 5% of the sample size at Haramaya General Hospital which found in Haramaya city to see the applicability of the instruments, and feedback was incorporated into the final tool to improve the quality. The result was not included in the results of this study. Supervisors and the principal investigator checked daily for completeness and consistency of the collected data. Codes were given for the completed questionnaires. Double data entry and verification was done to avert any error to happen during data entry.

### Measurements

Non-adherence is defined as a patient’s failure to follow the specified treatment plan, which may include medication, lifestyle modifications, or other suggestions from mental health care professionals [28].

Schizophrenia is a clinical diagnosis reached by the clinician based on DSM V diagnostic criteria. The presence of two or more of the following symptoms: delusion, hallucinations, disorganized speech, and behaviors with impaired social or occupational functions at least for the last 6 months is required [29].Medication adherence rating scale (MARS) scores less than or equal to 2 indicate non-adherence, while scores of 3 or above indicate adherence [25].

Substance use refers to using any psychoactive substances at least once during the last 3 months A family history of mental illness refers to the presence of any mental disorders among an individual’s first or second-degree relatives or a family member diagnosed with any mental illness, or a diagnosis of a specific mental illness in a family member The types of medication were assessed from medical records (cards).

### Statistical analysis

The collected data was checked for completeness and consistency, coded and double-entered, validated and cleaned using EpiData 3.1, and analyzed using STATA 14.1. The descriptive statistics of the categorical data were summarized using frequency percentage, mean, and standard deviation. Variables with p-values less than 0.25 in the bivariate analysis were considered in the multivariable analysis. Using multivariable logistic regression, the relationship between the independent and dependent variables was identified. Multiple stepwise models were used to explore the relationship between various factors and nonadherence to antipsychotic medications. Model 1 depicts the relationship between sociodemographic variables and non-adherence to antipsychotic medication. Model 2 depicts the relationship between substance use and nonadherence. Model 3 depicts the association between clinical and patient-related variables and nonadherence. Model 4 is the final model that shows the relationship between all independent variables and non-adherence in schizophrenia patients. The final result was reported using an odds ratio and a 95% confidence interval. Finally, a p-value of < 0.05 was considered a statistically significant association.

### Ethical considerations

Ethical clearance was obtained from the Institutional Health Research Ethical Review Committee (IHRERC) of the College of, Health and Medical Sciences, Haramaya University (Ref.No. IHRERC/255/2020). A copy of the ethical letter was submitted to the hospital administration. A letter of cooperation was written to the respective psychiatric department of the Hospital. Consent to participate in the study was obtained from, all study participants before the data collection. The informed, voluntary, written, and signed consent was obtained from each, participant. In addition, informed, voluntary, written, and signed, assent was also obtained from the caretakers and parents of, schizophrenia patients. Study participants were informed, about their right to withdraw from the study at any stage. Confidentiality of the information was assured. The data collectors interviewed the participants in separated room.

## Results

Out of the 911 individuals with schizophrenia recruited, 893 completed the questionnaire fully, giving a 98% response rate. The mean age was 34.2 years old, with a standard deviation of 10.39 years. The majority of the respondents 729(81.63%) were male sex, 508(56.89%) were from urban areas, 335(37.51%) were in the age category of 25–34, 359 (40.20%) were single marital status, 427 (47.82%) had no formal education, and 416(46.58%) were jobless.

**Table 1:** Socio-demographic characteristics of the study participants at public hospitals eastern Ethiopia from December 1, 2022, to January 31, 2023

Characteristics	Categories	(*N* = 893)	
		Frequency	%
Gender	Male Female	729 164	81.63 18.37
Residence	Urban Rural	508 385	56.89 43.11
Age in years	18–24 25–34 35–44 >=45	138 335 287 133	15.45 37.51 32.14 14.89
Marital status	Married Single Separated/Divorced	274 359 260	30.68 40.20 29.12
Education	High school and above Primary level No formal education	207 259 427	23.18 29.00 47.82
Occupation	Employed Daily labor Jobless Other	199 188 416 90	22.28 21.05 46.58 10.08

### Substance use behavior, clinical and patient-related and the prevalence of non-adherence among patients with schizophrenia

Substance use was more common among patients with schizophrenia who were non-adherence to their antipsychotic medication. Three in every eight (36.95%) of study participants had a history of lifetime tobacco use, while 402 (45.02%) were current cigarette smokers, 222 (55.22) were non-adherence to their medications, and 340 (38.1%) consumed alcohol, 188 (55.29) were non-adherence to their medications.

Over 51% of the participants (463 (51.9%) chewed Khat in the last 30 days of the data collection period, 255 (55.08%) of them were non-adherence to their medication, 412 (46.14) use more than one psychoactive substance (poly substance users), and 222 (53.88%) of poly substance users were non-adherence, while 215 (24.08%) of the respondents have problems with their family due to substance use habit, Two-fourths (49.72%) of patients with schizophrenia have a family history of mental illness, and four out of seven (56.77) participants have illness duration for more than five years. Additionally, 406 (45.46) of them have been on antipsychotic treatment for more than three years, and 400 (44.79) have been treated with first-generation antipsychotics, and 198(49.50) of them are non-adherence to their medication, whereas 403 (45.13) have no insight into their illness and 204(50.62%) of them non adherence. Two in every seven (28.56%) have antipsychotic medication-related severe side effects, and 135 (52.94%) of them have non-adherence to their medication.

### Bivariate logistic regression analysis was used

Bivariate analysis results revealed that single marital status, current substance use such as cigarette smokers, smokeless tobacco products, alcohol consumption, khat chewing, poly substance use, family history of mental illness, shorter duration of illness, no insight into their illness, being treated with first generation antipsychotics and treated with poly pharmacy, and the presence of antipsychotic related side effects were factors significantly associated with non-addiction.

**Table 2:** Substance use characteristics, Clinical and patient-related and rate of non-adherence with Chi-squared tests results among study participants at public Hospitals, eastern Ethiopia from December 1, 2022, to January 31, 2023

Characteristics	Categories	(*N* = 893) frequency (%)	Adhered 495 (55.43%)	Non-adhered398 (44.57%)	chi2(Pr)
Gender	Male Female	729 (81.63) 164(18.37)	405(55.56) 90(54.88)	332(44.44) 74(45.12)	Pr = 0.875
Age *	18–24 25–34 35–44 >=45	138 (15.45) 335 (37.51) 287 (32.14) 133(14.89)	76 (55.07) 204(60.90) 151(52.61) 64(48.12)	62(44.93) 131(39.10) 136(47.39) 69(51.88)	Pr = 0.049
Residency	Urban Rural	508 (56.89) 385 ( 43.11)	283(55.71) 212(55.06)	225(44.94) 173(44.94)	Pr = 0.848
Marital status *	Married Single Separated/Divorced	274(30.68) 359(40.20) 260 (29.12)	174(63.50) 152(42.34) 169(65.0)	100(36.50) 207(57.66) 91(35.00)	Pr = 0.000 )
Education	High school and above Primary level No formal education	207( 23.18 ) 259 (29.00) 427 ( 47.82)	116 (56.04 ) 137 (52.90) 242( 56.67)	91 (43.96) 122 (47.10 ) 185 (43.33)	Pr = 0.615
Occupation	Employed Daily labor Jobless Other	199 (22.28 ) 188 ( 21.05) 416 (46.58 ) 90 ( 10.08)	113 (56.78 ) 97 (51.60) 235 ( 56.49 ) 50(55.56 )	86 (43.22) 91(48.40) 181(43.51) 40(44.44 )	Pr = 0.692
Lifetime tobacco use	No Yes	563 ( 63.05) 330 ( 36.95)	312(55.42) 183(55.45)	251(44.58) 147(44.55)	Pr = 0.991
Cigarette smokers*	No Yes	491 ( 54.98) 402 ( 45.02)	315(64.15) 180(44.78)	176(35.85) 222(55.22)	Pr = 0.000
Smock less tobacco use*	No Yes	642( 71.89 ) 251(28.11)	378(58.88) 117(46,61)	264(41.12) 134(53.39)	Pr = 0.001
Alcohol consumption*	No Yes	553 (61.93) 340( 38.07)	343(62.03) 152(44.71)	210(37.97) 188(55.29)	Pr = 0.000
Current Khat use*	No Yes	430( 48.15 ) 463(51.85)	287(66.74) 208(44.92)	143(33.26) 255(55.08)	Pr = 0.000
Poly substance use	No Yes	481 (53.86) 412(46.14)	305(63.41) 190(46.12)	176(36.39) 222(53.88)	Pr = 0.000
Family history of mental health problem *	No Yes	449 (50.28) 444 (49.72)	268(59.69) 227(51.13)	181(40.31 217(48.87)	Pr = 0.010
Family history of substance use*	No Yes	460 (51.51) 433 (48.49)	277(60.22) 218(50.35)	183(39.78) 251(49.65)	Pr = 0.003
Illness durations*	> 5 years ≤ 5years	507 (56.77) 386 ( 43.23)	315(61.93) 181(46.89)	193(38.07) 205(53.11)	Pr = 0.000
Insight to their illness*	Have insight No insight	490 (54.87) 403(45.13)	296(60.41) 199(49.38)	194(39.59) 204(50.62)	Pr = 0.001
Medication type *	Second-generation First generation polypharmacy	240 (26.88) 400 (44.79) 253 (28.33)	155(64.58) 202(50.50) 138(54.55)	85(35.42) 198(49.50) 115(45.45)	Pr = 0.002 )
Antipsychotic medication side effect*	No side effect Mild side effect sever side effect	323 (36.17) 315 (35.27) 255 (28.56)	196(60.68) 179(56.83) 120(47.06)	127(39.32) 136(43.17) 135(52.94)	Pr = 0.004

### Prevalence of antipsychotic medication non-adherence and reasons for non-adherence among patients with schizophrenia

The overall prevalence of antipsychotic medication non-adherence was 398 (44.57%). The most common reason for non-adherence are Forgetting 470 (52.63%), taking medicine only while sick 446 (49.94%), and discontinuing when feeling better 385 (43.11%).

**Fig. 1:**
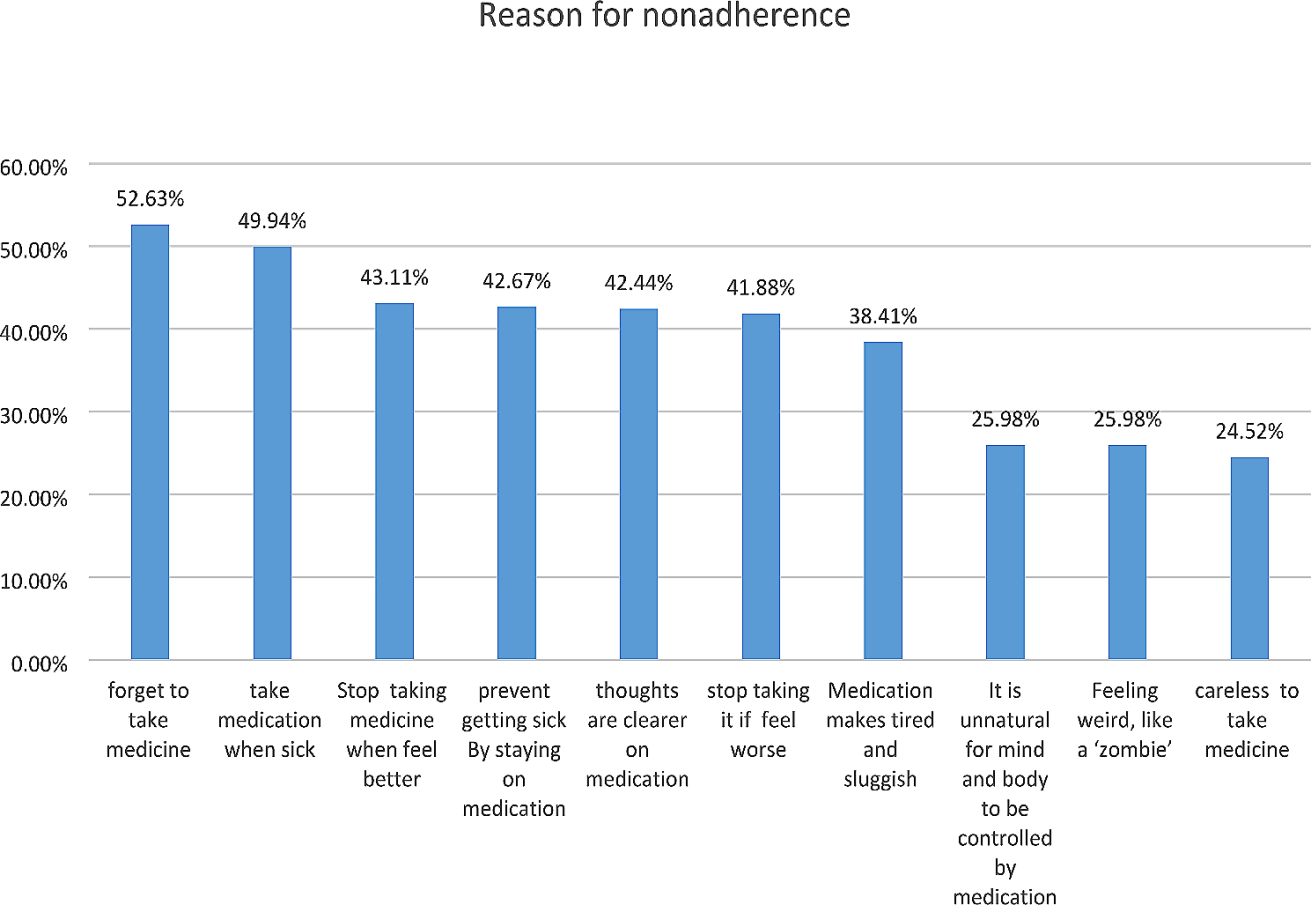
Reason for non-adherence among with schizophrenia at public Hospitals, eastern Ethiopia from December 1, 2022, to January 31, 2023

### Factors associated with antipsychotic medication non-adherence

In the bivariate logistic regression analysis, single marital status current substance usage such as cigarette smokers, smokeless tobacco products, alcohol use, khat chewing, Poly substance use, family history of mental illness, shorter duration of illness, no insight into their illness, being treated with first generation antipsychotics and treated with poly pharmacy, and the presence of antipsychotic related side effects were factors significantly associated with medication non-adherence among patients with schizophrenia at p 0.25, but gender, age, educational status, and participant occupation were not associated.

Single marital status, current substance use such as smoking, chewing, or drinking alcohol, a family history of mental illness, being treated with first-generation antipsychotic medication, being treated with polypharmacy or more than one antipsychotic medication, and the presence of severe side effects were all significantly associated with medication non-adherence in the multivariable logistic regression.

The odds of non-adherence to antipsychotic medication were 2.3 times higher (AOR 2.37; 95% CI (1.670–3.365) among single individuals compared to those who are married; (model 1), 4 times higher (AOR 4.278; 95% CI (1.455–12.57) among current cigarette smokers compared to non-smokers; and 2.6 times higher (AOR = 2.698; 95% CI (1.938–3.765) among current khat chewers compared to non-chewers; (model 2), Whereas 1.3 times higher (AOR = 1.377;95% CI: 1.043–1.820) among those who have a family history of mental illness, 1.7 times higher (AOR = 1.719; 95% CI: 1.219–2.426) among those who are treated with first-generation antipsychotics compared with those treated with second-generation antipsychotics, and 2 times higher (AOR = 2.04;95% CI: 1.420–2.946) among those who have severe side effects compared to their counterpart.

(Model 3). In the final model 4, all variables and non-adherence were regressed together. The findings revealed that non-adherence to medication was found to be two times higher among single marital status (AOR = 2.478; 95% CI(1.713–3.855) compared to married, 2 times higher (AOR = 2.034; 95% CI (1.496–2.674) among current cigarette smoker compared to non-smokers, 1.9 times more common (AOR = 1.991; 95% CI (1.457–2.719) among current alcohol users to non-alcohol users, 2.8 times more common (AOR = 2.836; 95% CI (2.0601–3.902) among current Khat chewers compared to non Khat chewers,1.4 times common (AOR 1.446; 95% CI (1.049–1.993) among those whose illness duration less than or equal to 5 years compared to their counterpart, 1.7 and 1.9 times higher (AOR = 1.950; 95% CI (1.267- 3.000) and (AOR = 1.760; 95% CI (1.204–2.573)among those who are on first generation and those who are on poly pharmacy antipsychotic compared to those on second generation antipsychotic, respectively, 2.1 times more common (AOR = 2.101; 95% CI (1.517–2.911) among those who have no insight to their illness compared with their counterpart, and 1.6 times higher (AOR = 1.654; 95% CI (1.103–2.482) among those who have medication side effect compared those not complain side effect (Table 3).

**Table 3:** Factors associated with antipsychotic medications non adherence. Among patients with schizophrenia at public Hospitals, Eastern Ethiopia from December 1, 2022, to January 31, 2023

Variable	Categories	Model 1	Mode2	Mode3	Mode 4
		AOR (95% CI)& Pv	AOR (95% CI) and Pv	AOR (95% CI) &Pv	AOR (95% CI)& Pv
Gender	Female male	Ref 0.9377(0.648- 1.355) Pv = 0.742			Ref 1.036(0.704-1.525)Pv = 0.858
Age	18–24 25–34 35–44 ≥ 45	Ref 0.754(0.498- 1.1409) Pv = 0.181 0.9983(0.651- 1.531) Pv = 0.998 1.156(0.701- 1.908) Pv = 0.561			Ref 0.691(0.439- 1.088) Pv = 0.111 0.842(0.523- 1.355) Pv = 0.479 0.855(0.497–1.473) Pv = 0.583
Marital status	Married Single Separated or divorced	Ref 2.371(1.670–3.365) Pv = 0.0001 0.951(0.653- 1.387) Pv = 0.795			Ref 2.478 (1.713–3.585) Pv= 0.0001 1.122(0.7497 − 1.680) Pv = 0.574
Current Cigarette smokers	No Yes		Ref 4.258(1.445–12.53)Pv = 0.009		Ref 2.034(1.496–2.764) Pv = 0.0001
Smock less tobacco use	No Yes		Ref 1.95 (1.41–2.71) Pv = 0.0001		2.168(1.521–3.091) Pv = 0.0001
Alcohol consumption	No Yes		Ref 1.89 (1.41–2.54) Pv = 0.0001		Ref 1.99(1.457–2.719) Pv = 0.0001
Current Khat use	No Yes		Ref 2.66(1.99–3.56) Pv = 001		Ref 2.836(2.0601–3.902) Pv = 0.0001
Poly substance use	No Yes		Ref 0.491(0.16–1.44) Pv = 0.197		
Family history of substance use	No Yes		Ref 1.38 (1.04–1.84) Pv = 0.025		Ref 2.663(0.465- 15.228) Pv= 0.271
Family history of mental health problem	No Yes			Ref 1.377 (1.043–1.820) Pv = 0.024	Ref 0.480(0.084- 2.743) Pv = 0.409
Illness durations	> 5 years ≤ 5years			Ref 1.820 (1.368–2.420) Pv = 0.001	Ref 1.446(1.049–1.993) Pv = 0.024
Insight to their illness	Have insight No insight			Ref 2.053 (1.525–2.764) Pv = 0.0001	Ref 2.101(1.517–2.911) Pv = 0.0001
Medication type	Second-generation First generation polypharmacy			Ref 1.719(1.219–2.4260) Pv = 0.002 1.816(1.227–2.688)Pv = 0.003	Ref 1.760(1.204–2.573) Pv = 0.004 1.950 (1.267-3.00) Pv = 0.002
Antipsychotic medication side effect	No side effect Mild side effect severe side effect			Ref 1.363 (0.9731 − 1.910)Pv = 0.072 2.045 (1.420–2.946)Pv = 0.000	Ref 1.299(0.8954 − 1.886) Pv = 0.168 1.654(1.103–2.482) Pv = 0.015

## Discussion

The primary treatment for patients with schizophrenia is antipsychotic medication, and taking it on a regular basis is critical for controlling symptoms and preventing relapses. According to our findings, the prevalence of non-adherence among patients with schizophrenia is 44.57% (95% CI: 0.41–0.47), our study found similar rates of non-adherence as studies done in Switzerland (44.8%) [30], but higher than non-adherence rates reported in England (29%) [31] and Jima Southwest Ethiopia (41.2%), [32] and lower than study from Egypt (74%), and Gondar Ethiopia(75.7%), [33, 34]. This could be explained by discrepancies in medication adherence measurement and sample size [30].

While it is clear that a variety of factors contribute to non-adherence in patients with schizophrenia, this might be due to higher substance use among schizophrenia patients, which may be one of the factors contributing to the high rate of non-adherence to their medication in our study setting. Except for single marital status, no socio-demographic factors were found to be associated with antipsychotic medication non-adherence during the bivariate analysis. This is in line with previous studies [10, 35].

According to our study findings, the most relevant associated factors include single marital status, current substance use, medication side effects, short duration of illness, and poor insight into their illness. Our study found no association between medication non-adherence and individuals’ gender, age, place of residence, educational status, or employment. Our result is consistent with previous studies [36], but contradicts the other previous study [37]. This could be due to differences in study population study setting and sample size.

According to the findings of our study, single marital status was 2.47 times non-adherence to their medications compared to married participants. This could be due to the ability of husband or wife partnerships to monitor or encourage medication use. A single person, on the other hand, may have difficulties getting financial assistance for hospital visits and treatment costs [38]. One possible solution could be to provide these individuals with additional resources, such as transportation services or home visits from mental health professionals, encourage these individuals to participate in support groups or other community-based programs that can provide them with the social support they need to manage their illness [39].

According to our study results, current substance use, such as cigarette smoking, drinking alcohol, and chewing, was significantly associated with nonadherence to antipsychotic treatment, which is consistent with the findings of other studies [3]. The relationship between substance use and non-adherence in patients with schizophrenia is complex and multifaceted [44].

Patients who are non-adherent to their medications are more likely to use substances as a form of self-medication. This is because non-adherence to medication can lead to a worsening of symptoms, which can cause distress and discomfort. Substance use can provide temporary relief from these symptoms, leading to a vicious cycle of non-adherence and substance use. It is important for physicians and other health professionals to understand the reasons behind non-adherence and substance use in order to develop effective interventions to improve patient outcomes [3, 40].

. Our findings show that more than half (55.08%) of participants who Chewing Khat did not take their medication as prescribed, which is consistent with other study findings [41]. This could be Eastern Ethiopia has the most khat cultivation for both export and domestic use, which may influence khat chewing habits. Khat chewing is a widespread and culturally accepted practice, and the vast majority of people engage in it [42, 43]. Chewing khat provides energy and euphoria, and it is also used to relax, improve concentration and waste time [3, 41].

The current study found that non-adherence was substantially associated with antipsychotic medication side effects this is inline with former study [45]. This is because the existence of side effects that were so distressing and associated with a considerable deterioration in their quality of life, which considered as more difficult than illness, hampered the people’ capacity to do some activities of daily living. This can have a negative impact on a person’s well-being and lower their quality of life [46].

According to our findings, antipsychotic medication non-adherence was substantially associated with poor or no insight to their illness. Our result is in line with previous studies from elsewhere [45, 47]. One possible explanation is that understanding of the illness and its treatment is important in medication adherence. Poor insight can range from absolute denial of illness and rejection of the precise diagnosis to symptom, mitigation and explanations, as well as a failure to recognize that medications are required to treat specific symptoms and lower the risk of relapse [22]. They avoided taking medication because they refused to admit that they were mentally ill and persistently non-adherence to their medication [40].

Moreover, a shorter duration of illness (≤ 5 years) was significantly associated with non-adherence, which was confirmed by another study conducted elsewhere [3, 46]. The possible explanation is that when an illness is short in duration, patients may have a poor understanding of their illness and the need for treatment, which leading to a negative attitude toward their medication, this negative attitude may result in non-adherence to prescribed medications [48].

### Strength of the study

As to our knowledge, this study is possibly the first study to assess the level and factors associated with antipsychotic medication non-adherence among patients with schizophrenia in eastern Ethiopia.

### Limitation of the study

Because of the cross-sectional design, we were unable to establish a causal relationship between significantly associated variables and antipsychotic medication non-adherence, as well as a lack of objective measures to measure medication adherence such as pill count or blood level estimation. Pill counts could not be done because most patients do not come to the follow-up clinic with pill boxes or medicine strips unless informed beforehand, and because this was a cross-sectional study, there was no way to know if there was a causal relationship.

## Conclusions

The purpose of this study was to assess antipsychotic medication non-adherence and factors associated among patients with schizophrenia in eastern Ethiopia. Thus, adherence to antipsychotic medication was a major issue among patients with schizophrenia, non-adherence to medications increases the, risk of exacerbating symptoms, relapse rate, increasing frequency of hospital visits and the burden of care. The most important factors for increased non-adherence were single marital status, substance use, and short duration of illness, no insight to the illness, taking first generation and more than one antipsychotic medication, and the presence of severe medication side effects. Reducing the number of antipsychotic medications and side effects while increasing insight or awareness about illness and the need for treatment may reduce non-adherence. Furthermore, clinicians should emphasize the importance of screening patients for substance abuse and providing brief motivational interventions to those in need of addiction rehabilitation services, which may lead to better adherence, this require the participation of all relevant stakeholders.

## Data Availability

The original contributions presented in the study are included in the article.

## Abbreviations

ASSIST: Alcohol, Smoking and Substance Involvement Screening Test
WHO: World health organization
